# Uniform and Anisotropic Solid Electrolyte Membrane Enables Superior Solid‐State Li Metal Batteries

**DOI:** 10.1002/advs.202100899

**Published:** 2021-06-02

**Authors:** Zumin Guo, Yuepeng Pang, Shuixin Xia, Fen Xu, Junhe Yang, Lixian Sun, Shiyou Zheng

**Affiliations:** ^1^ School of Materials Science and Engineering University of Shanghai for Science and Technology Shanghai 200093 China; ^2^ Guangxi Key Laboratory of Information Materials & Guangxi Collaborative Innovation Centre of Structure and Property for New Energy and Materials Guilin University of Electronic Technology Guangxi 541004 China

**Keywords:** anisotropic conduction, coaxial electrospinning, solid composite electrolyte, solid‐state Li batteries, uniform dispersion

## Abstract

Rational structure design is a successful approach to develop high‐performance composite solid electrolytes (CSEs) for solid‐state Li metal batteries. Herein, a novel CSE membrane is proposed, that consists of interwoven garnet/polyethylene oxide‐Li bis(trifluoromethylsulphonyl)imide (LLZO/PEO‐LiTFSI) microfibers. This CSE exhibits high Li‐ion conductivity and exceptional Li dendrite suppression capability, which can be attributed to the uniform LLZO dispersion in PEO‐LiTFSI and the vertical/horizontal anisotropic Li‐ion conduction in the CSE. The uniform LLZO particles can generate large interaction regions between LLZO and PEO‐LiTFSI, which thus form continuous Li‐ion transfer pathways, retard the interfacial side reactions and strengthen the deformation resistance. More importantly, the anisotropic Li‐ion conduction, that is, Li‐ion transfers much faster along the microfibers than across the microfibers, can effectively homogenize the electric field distribution in the CSE during cycling, which thus prevents the excessive concentration of Li‐ion flux. Finally, solid‐state Li||LiFePO_4_ cells based on this CSE show excellent electrochemical performances. This work enriches the structure design strategy of high‐performance CSEs and may be helpful for further pushing the solid‐state Li metal batteries towards practical applications.

## Graphical abstract

A novel composite solid electrolyte that consists of interwoven inorganic/organic hybrid microfibers is proposed, which exhibit uniform inorganic filler dispersion and vertical/horizontal anisotropic Li‐ion conduction. These unique characteristics enable superior Li‐ion conduction properties, especially in terms of even Li plating due to the homogenization of the electric field distribution in the electrolyte.

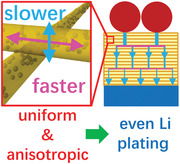

## 1 Introduction

Rechargeable Li metal batteries with high energy density, long cycling life, and excellent safety are extensively explored for the widespread application of clean and renewable energy sources in electric vehicles and smart grids.^[^ ^1^, ^2^, ^3^, ^4^ ^]^Replacement of liquid electrolytes by solid electrolytes is recently a research hotspot, because i) they are non‐vaporizable and nonflammable, which avoids the fire and explosion accident under extreme conditions, and ii) they potentially have better electrochemical stability and mechanical strength, which enables the use of Li anode and aggressive cathodes.^[^ ^5^, ^6^, ^7^, ^8^, ^9^, ^10^, ^11^ ^]^

Solid electrolytes can be classified into two categories: inorganic electrolytes and polymer electrolytes. Inorganic electrolytes usually exhibit attractive Li‐ion conductivities, high mechanical moduli, and wide electrochemical stability windows, but their rigidness and brittleness seriously decrease the electrode compatibility.^[^ ^12^, ^13^ ^]^ Polymer electrolytes are more flexible and viscoelastic, which can easily form well‐contacted electrolyte/electrode interfaces, but they suffer from low Li‐ion conductivity, poor mechanical strength, and bad electrochemical stability.^[^ ^14^, ^15^ ^]^

The strategy of forming composite solid electrolytes (CSE) by incorporating inorganic electrolytes into polymer electrolyte matrix is very promising that can not only improve ionic conductivity, but also enhance the mechanical strength without sacrificing flexibility.^[^ ^16^, ^17^, ^18^, ^19^, ^20^, ^21^, ^22^, ^23^, ^24^, ^25^, ^26^ ^]^ As representative materials, garnet/polyethylene oxide‐lithium bis(trifluoromethylsulphonyl)imide (LLZO/PEO‐LiTFSI) CSEs show high conductivity and excellent Li dendrite suppression capability.^[^ ^27^ ^]^

Extensive efforts have been committed to the structural design of the LLZO/PEO‐LiTFSI CSEs for achieving uniform LLZO dispersion in PEO‐LiTFSI matrix, which can i) generate large and continuous LLZO/PEO‐LiTFSI interface, resulting in an improved Li‐ion conductivity and ii) enhance the mechanical properties of the CSEs, leading to a high Li dendrite suppression capability.^[^ ^28^, ^29^ ^]^ Huo et al.^[^ ^30^ ^]^ reported that the particle size of LLZO is a key factor, in which 0.2 µm LLZO can increase the ionic conductivity of the LLZO/PEO‐LiTFSI CSE and 5 µm LLZO can enhance the tensile strength of the LLZO/PEO‐LiTFSI CSE. Fu et al.^[^ ^31^ ^]^ designed a 3D LLZO nanofiber network/PEO‐LiTFSI CSE, which exhibits continuous Li‐ion transfer channels and can enable stable cycling of Li symmetric cells. Li et al.^[^ ^32^ ^]^ prepared a 3D LLZO sponge framework/PEO‐LiTFSI CSE, in which the interconnected LLZO provides continuous ionic transport pathways and strengthens the mechanical properties.

Although the above pioneering works have made great progress, further exploration of novel LLZO/PEO‐LiTFSI CSEs with more optimized structures is still highly needed to meet the practical requirements, especially in terms of Li‐ion conductivity and Li dendrite suppression capability.

Recently, pioneering investigations proposed that high vertical Li‐ion conductivity can be achieved by vertically aligned inorganic fillers (such as vermiculite^[^ ^33^ ^]^ and Li_1.5_Al_0.5_Ge_1.5_(PO_4_)_3_^[^ ^34^, ^35^ ^]^) or vertically aligned porous hosts (such as polyimide^[^ ^36^ ^]^ and Al_2_O_3_^[^ ^37^ ^]^). This inspired us that Li‐ion conduction anisotropy is another key factor that significantly affects the overall performances of CSEs.^[^ ^38^ ^]^

Electrospun membranes exhibit a unique structure of interwoven microfibers.^[^ ^39^, ^40^ ^]^ We expect that this unique structure can effectively generate uniform filler dispersion and vertical/horizontal anisotropy. In this work, a CSE membrane that consists of interwoven LLZO/PEO‐LiTFSI microfibers is prepared by the coaxial electrospinning method. This CSE shows uniform LLZO dispersion and vertical/horizontal anisotropic Li‐ion conduction due to the unique structure, which enables the high Li‐ion conductivity and exceptional Li dendrite suppression capability. In addition, solid‐state Li||LiFePO_4_ cells based on this CSE demonstrate superior overall electrochemical performances, suggesting a promising application prospect.

## 2 Results and Discussions

**Figure 1:**
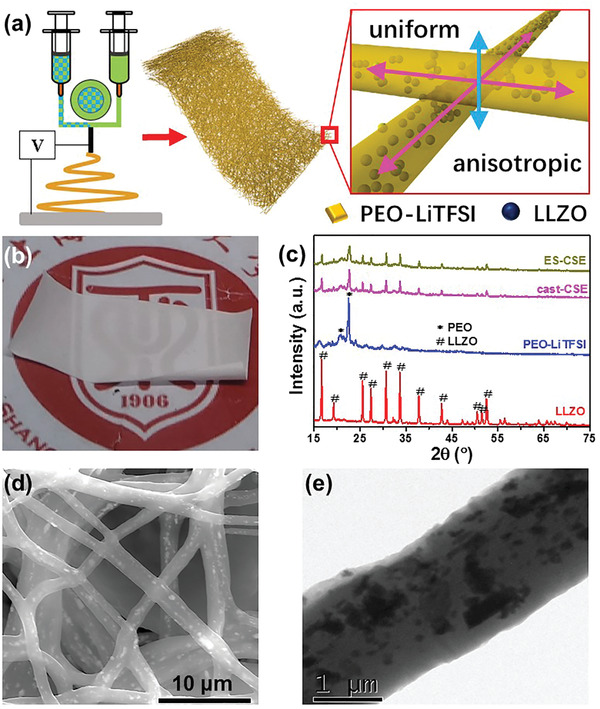
Preparation and structure characterizations. a) Schematic illustration of the design principle of ES‐CSE. b) Optical photograph of ES‐CSE. c) XRD patterns of LLZO, PEO‐LiTFSI, cast‐CSE, and ES‐CSE. d) Top‐view SEM image of ES‐CSE. e) TEM image of ES‐CSE.

As seen in **Figure** **1a**, an electrospun composite solid electrolyte membrane (ES‐CSE) is prepared by coaxial electrospinning with PEO/acetonitrile (ACN) as shell solution and LLZO/PEO/LiTFSI/ACN as core solution, which is then followed by drying to completely remove ACN. The as prepared membrane is expected to be composed of uniform and anisotropic interwoven LLZO/PEO‐LiTFSI microfibers, which may be of great help for its overall performances. Optical photograph (Figure 1b) shows that ES‐CSE is thin and flexible, matching well with the requirements of electrolytes for both coin and pouch cells.

Figure 1c displays the X‐ray diffraction (XRD) patterns of ES‐CSE and reference samples. Tape casting composite solid electrolyte membrane (cast‐CSE) with the same composition exhibit typical peaks of LLZO and PEO‐LiTFSI, indicating no side reaction. As for ES‐CSE, the diffraction peaks of PEO‐LiTFSI become much weaker, which suggests that the crystallinity degree of PEO is decreased, benefiting the Li‐ion transfer through polymer chain segment movement.^[^ ^41^ ^]^

Scanning electron microscope (SEM) and transmission electron microscope (TEM) images (Figure 1d,e) of ES‐CSE present that LLZO particles with a diameter of 100 to 500 nm are uniformly dispersed in interwoven PEO‐LiTFSI microfibers with a diameter of 1.46 ± 0.32 µm (Figure S1, Supporting Information). As a comparison, an apparent particle agglomeration is observed for cast‐CSE (Figure S2, Supporting Information). The uniform dispersion can be attributed to the confinement of LLZO particles within the PEO‐LiTFSI microfibers through the unique coaxial electrospinning method. The porosity of ES‐CSE is estimated to be 25% (Figure S3, Supporting Information). In addition, Fourier transform infrared (FTIR) spectra (Figure S4, Supporting Information) show that the dispersed LLZO can stimulate the interaction between PEO and TFSI^−^.^[^ ^17^ ^]^ The thickness of ES‐CSE can be easily tailored by controlling the electrospinning duration. For example, 135 µm for 5 h and 200 µm for 8 h (Figure S5, Supporting Information).

**Figure 2:**
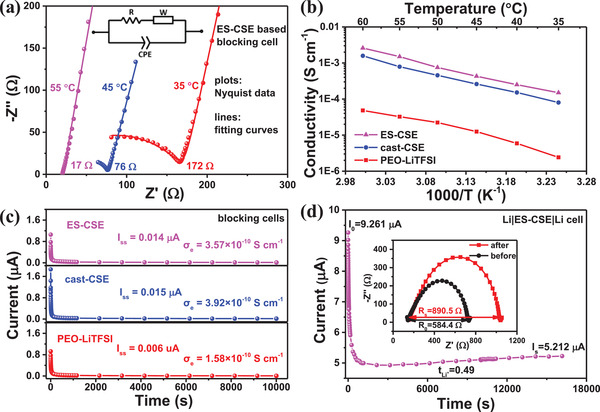
Li‐ion conduction properties. a) EIS of ES‐CSE at 35, 45, and 55 °C. b) Temperature‐dependent conductivities of PEO‐LiTFSI, cast‐CSE, and ES‐CSE. c) electronic conductivities of PEO‐LiTFSI, cast‐CSE, and ES‐CSE at 55 °C. d) Li‐ion transference numbers of ES‐CSE at 55 °C.

The Li‐ion conduction properties of ES‐CSE are systematically verified with PEO‐LiTFSI and cast‐CSE as reference samples. The resistances of the electrolytes are measured using electrochemical impedance spectra (EIS) of blocking cells, and representative results are shown in **Figure** **2a**. Each Nyquist plots consist of a semicircle at high frequency and a linear tail at low frequency, in which the diameter of the semicircle corresponds to the resistance of the electrolyte (inset of Figure 2a). Then, the conductivity (*σ*) of the electrolyte can be calculated according to

(1) $$\sigma =\frac{d}{A\cdot R}$$

where *d* is the thickness, *A* is the contact area, and *R* is the resistance. The temperature‐dependent conductivities of PEO‐LiTFSI, cast‐CSE, and ES‐CSE are displayed in Figure 2b. ES‐CSE demonstrates the highest conductivity, as it reaches 1.5 × 10^−4^ S cm^−1^ at 35 °C and 1.5 × 10^−3^ S cm^−1^ at 55 °C, which are 58 and 2 times higher than those of PEO‐LiTFSI and cast‐CSE, respectively. It is noteworthy that the LLZO content in ES‐CSE is optimized to be 10.8 wt% (Figure S6, Supporting Information). The improvement in conductivity can be attributed to the highly uniform dispersion of LLZO in the PEO‐LiTFSI matrix.

The direct current (DC) polarization measurement (Figure 2c) of the blocking cell at 55 °C shows that the electronic conductivity of ES‐CSE is in the order of 10^−10^ S cm^−1^, corresponding to a neglectable electronic transference number, which is similar to those of the PEO‐LiTFSI and cast‐CSE. The Li‐ion transference number of ES‐CSE is calculated to be 0.49 according to the DC polarization and EIS measurements (Figure 2d) of the Li symmetrical cell, which is 2.1 and 1.6 times higher than those of PEO‐LiTFSI (0.23) and cast‐CSE (0.30), respectively (Figure S7, Supporting Information). These results confirm that the uniform LLZO dispersion in the PEO‐LiTFSI matrix can enhance the Li‐ion transference number without sacrificing electronic resistance.

Therefore, we believe that the uniformly dispersed LLZO in ES‐CSE can not only promote the conductivity by generating continuous Li‐ion transfer pathways, but also increase the Li‐ion transference number by facilitating the immobilization of TFSI^−^.

**Figure 3:**
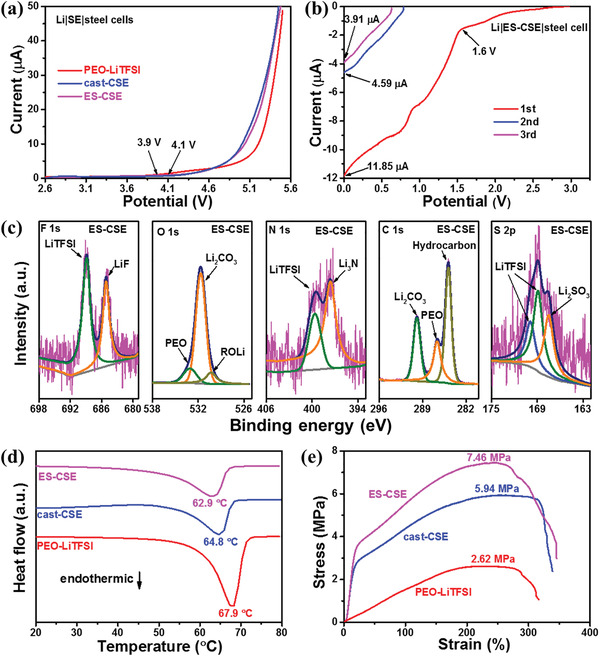
Stability measurements. a) LSV curves of PEO‐LiTFSI, cast‐CSE, and ES‐CSE from OCV to 5.5 V at 55 °C. b) Repeated LSV curves of ES‐CSE from OCV to 0 V at 55 °C. c) XPS results of the in situ formed SEI on the steel side for ES‐CSE after repeated scanning from OCV to 0 V. d) DSC curves of PEO‐LiTFSI, cast‐CSE, and ES‐CSE. e) Stress‐strain curves of PEO‐LiTFSI, cast‐CSE, and ES‐CSE.

Besides Li‐ion conduction properties, the electrochemical, thermal, and mechanical stabilities are also key factors for electrolytes. Linear sweep voltammetry (LSV) curves (**Figure** **3a**) of Li|electrolyte|steel cells from the open‐circuit voltage (OCV) to 5.5 V at 55 °C present the upper limits of the electrochemical stability windows for PEO‐LiTFSI, cast‐CSE, and ES‐CSE. The oxidation current starts to rise at 3.9 V for PEO‐LiTFSI, and apparent oxidation signals for cast‐CSE and ES‐CSE can be observed at above 4.1 V. As seen in the LSV curve (Figure 3b) from OCV to 0 V at 55 °C, the lower limit of the electrochemical stability window for ES‐CSE is 1.6 V, much higher than the working potential of Li anode. It is known that solid electrolyte interphase (SEI) acts an important role in improving the electrolyte/anode compatibility, so the LSV test from OCV to 0 V is repeated to clarify the SEI effect.^[^ ^42^, ^43^ ^]^ The maximum current is significantly reduced from 11.85 µA for the first cycle to 3.91 µA for the third cycle, indicating that the in situ formed SEI can prevent ES‐CSE from further electrochemical reduction. PEO‐LiTFSI and cast‐CSE also exhibit similar but worse electrochemical behaviors in the repeated LSV tests (Figure S8, Supporting Information) from OCV to 0 V. From the above results, we can deduce that the apparent electrochemical stability of PEO‐LiTFSI can be improved by LLZO incorporation, and higher dispersion leads to more effective retardation of side reactions.

The in situ formed SEI is then identified using X‐ray photoelectron spectroscopy (XPS). As shown in Fig. 3c, besides for PEO and LiTFSI from ES‐CSE, the signals for LiF, Li_3_N, Li_2_CO_3_, ROLi, and Li_2_SO_3_ can be observed in the spectra,^[^ ^44^, ^45^ ^]^ which suggest that both PEO and LiTFSI are electrochemically lithiated during the repeated LSV scanning from OCV to 0 V. We speculate that the LiF‐ and Li_3_N‐containing SEI can prevent the further side reaction at the electrolyte/electrode interface with allowing the Li‐ion conduction (Figure S9, Supporting Information). As a comparison, the XPS results of the in situ formed SEI for cast‐CSE are also provided in Figure S10, Supporting Information, in which a similar composition can be detected.

Differential scanning calorimetry (DSC) measurements (Figure 3d) are conducted to verify the thermal stabilities of PEO‐LiTFSI, cast‐CSE, and ES‐CSE. Only one endothermic peak can be found in each curve, corresponding to the melting of PEO.^[^ ^46^ ^]^ The melting temperatures for PEO‐LiTFSI, cast‐CSE, and ES‐CSE are 67.9, 64.8, and 62.9 °C, suggesting different degree of crystallinity of PEO, which agrees well with the XRD results. It should be noted that the melting temperature of ES‐CSE, though being decreased, is still high enough for practical use. Stress‐strain curves (Figure 3e) show that the tensile strength of ES‐CSE is 7.46 MPa, much higher than those of PEO‐LiTFSI (2.62 MPa) and cast‐CSE (5.94 MPa). This can be attributed to the mechanical strengthening effect of the uniformly dispersed LLZO and interwoven PEO‐LiTFSI microfibers. The improved mechanical stability of ES‐CSE provides safety reinforcement against battery abuse.

**Figure 4:**
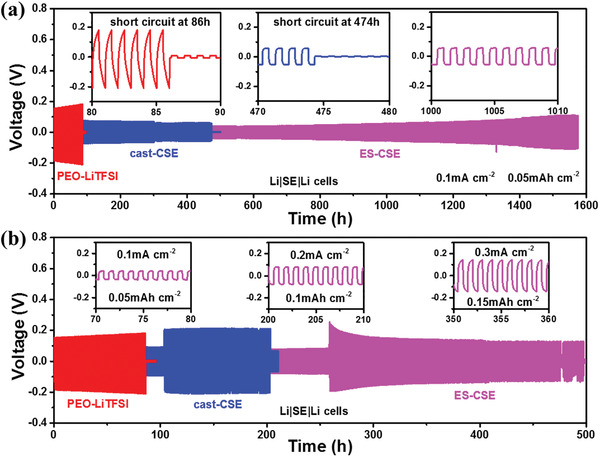
Li dendrite suppression capabilities at 55 °C. a) GCD profiles of Li symmetrical cells based on PEO‐LiTFSI, cast‐CSE, and ES‐CSE at 0.1 mA cm^−2^ and b) successive 0.1, 0.2, and 0.3 mA cm^−2^.

The Li dendrite suppression capabilities of PEO‐LiTFSI, cast‐CSE, and ES‐CSE are evaluated by galvanostatic charge and discharge (GCD) of Li symmetric cells. As shown in **Figure** **4a**, the ES‐CSE‐based cell shows a flat overvoltage of 50 mV in the initial 1000 h at 0.1 mA cm^−2^ at 55 °C and can maintain stable cycling for over 1500 h. For PEO‐LiTFSI and cast‐CSE, the overvoltages are sharper and higher (180 and 70 mv), and the cycling durations without short circuit are much shorter (86 and 474 h). It should be noted that ES‐CSE‐based Li symmetric cell can also stably cycle at 0.5 mA cm^−2^ and 55 °C for over 160 h as well as at 0.1 mA cm^−2^ and 35 °C for over 750 h (Figure S11, Supporting Information).

Figure 4b presents the GCD curves of Li symmetric cells at 0.1, 0.2, and 0.3 mA cm^−2^ at 55 °C. Increasing the current densities leads to sharper and higher overvoltages, and thus results in easier Li dendrite formation.^[^ ^47^ ^]^ ES‐CSE base Li symmetric cell successively exhibits stable Li stripping and plating on both sides at 0.1 mA cm^−2^ for 100 h, 0.2 mA cm^−2^ for 150 h, and 0.3 mA cm^–2^ for 250 h. While for PEO‐LiTFSI and cast‐CSE, short circuits occur at 0.1 mA cm^−2^ after 86 h cycling and 0.2 mA cm^−2^ after 203 h cycling, respectively.

The above results definitely reveal that the Li dendrite suppression capability is remarkably improved by forming the unique structure of interwoven LLZO/PEO‐LiTFSI microfibers. This can be ascribed to the enhancement in i) Li‐ion conduction properties to decrease the overvoltage, ii) electrochemical and mechanical stabilities to restrain the dendritic Li growth, and more importantly, iii) vertical/horizontal anisotropic Li‐ion conduction to homogenize the electric field distribution.

The vertical/horizontal anisotropic Li‐ion conduction in ES‐CSE is directly demonstrated by orientational Li stripping and plating measurements. It is known that electrolytes are Li‐ion conductors and electron insulators, so the resistance between two Li electrodes is inverse proportion to the Li‐ion conductivity according to Equation (1). To guarantee identical experimental conditions, all measurements are performed without packing.

**Figure 5:**
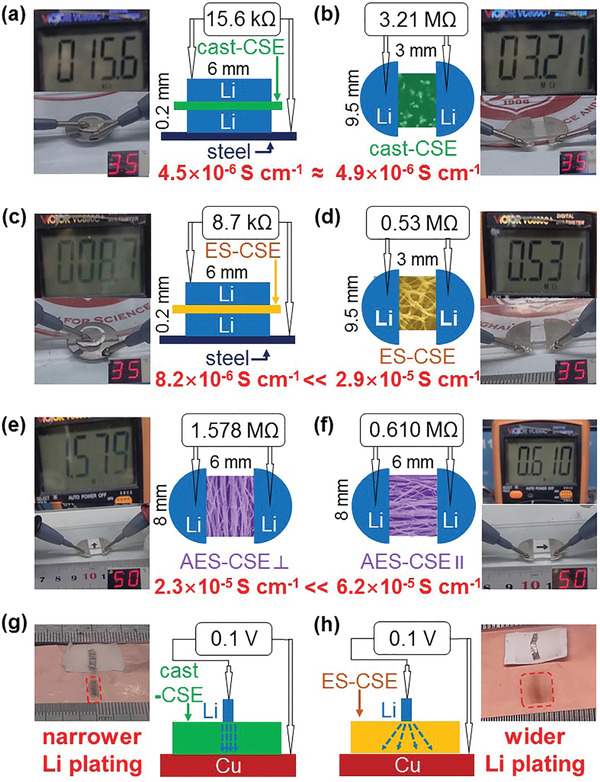
Vertical/horizontal anisotropic Li‐ion conduction experiments. a) Vertical and b) horizontal Li‐ion transfer resistances of cast‐CSE. c) Vertical and d) horizontal Li‐ion transfer resistances of ES‐CSE. Li‐ion transfer resistances of e) AES‐CSE⊥ and f) AES‐CSE∥. Electrochemical Li plating on Cu foil through g) cast‐CSE and h) ES‐CSE.

**Figure****5a** shows the vertical Li‐ion conductivity measurement of cast‐CSE, in which the cell structure is a cast‐CSE between two Li foils. The vertical Li‐ion conductivity is calculated to be 4.5 × 10^−6^ S cm^−1^ at 35 °C according to the thickness (0.2 mm), area (28.26 mm^2^), and resistance (15.6 kΩ). Figure 5b displays the horizontal Li‐ion conductivity measurement of cast‐CSE. Two semicircle Li foils are parallelly pressed on the same side of a rectangular cast‐CSE at a certain distance. The horizontal Li‐ion conductivity is calculated to be 4.9 × 10^−6^ S cm^−1^ at 35 °C according to the distance (3 mm), area (1.90 mm^2^) and resistance (3.21 MΩ), which is close to the vertical value, showing an isotropic nature.

The measurements of the vertical and horizontal Li‐ion conductivities of ES‐CSE are presented in Figure 5c,d, respectively. The cell structures are the same as those of cast‐CSE, while the horizontal Li‐ion conductivity (2.9 × 10^−5^ S cm^−1^) at 35 °C is 3.5 times larger than the vertical value (8.2 × 10^−6^ S cm^−1^). This huge difference indicates an anisotropic nature of ES‐CSE, which is mainly derived from the much more continuous Li‐ion transfer pathways in the horizontal orientation than that in the vertical orientation.

To further confirm the anisotropic Li‐ion conduction along and across the LLZO/PEO‐LiTFSI microfibers, the aligned electrospinning composite solid electrolyte membrane (AES‐CSE, Figure S12, Supporting Information) is prepared using a high‐speed rotating drum collector and investigated in terms of perpendicular (⊥) and parallel (∥) Li‐ion conductivity. As seen in Figure 5e,f, the Li‐ion conductivity along the microfibers is 2.7 times larger than that across the microfibers, proving the existence of the anisotropic Li‐ion conduction along and across the LLZO‐PEO‐LiTFSI microfibers. It should be noted that the experimental conditions of these horizontal Li‐ion conduction measurements are well controlled, ensuring the accuracy and reliability of the results (Figure S13, Supporting Information).

The anisotropic Li‐ion conduction in ES‐CSE is beneficial to the homogenization of electric field distribution and thus leads to uniform Li stripping and plating. Figure 5g,h shows the measurements of electrochemical Li plating from narrow Li foils to wide Cu foils, which simulate the inevitable occurrence of high local Li flux through the electrolyte during battery cycling. The Li plating area (black region) for ES‐CSE is wider than that for cast‐CSE and the Li plating morphology for ES‐CSE is more uniform than that for cast‐CSE (Figure S14, Supporting Information), which both provide direct proof for the effective horizontal Li‐ion transfer in ES‐CSE.

**Figure 6:**
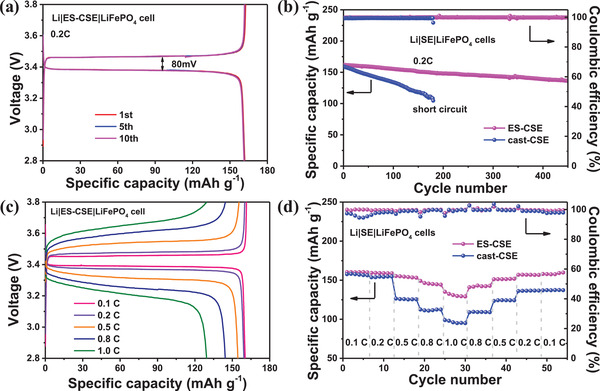
Electrochemical performances of solid‐state Li||LiFePO_4_ cells at 55 °C. a) GCD profiles for ES‐CSE at 0.2 C. b) Cycling performance for cast‐CSE and ES‐CSE at 0.2 C. c) GCD profiles for ES‐CSE at 0.1–1.0 C. d) Rate performances for cast‐CSE and ES‐CSE at 0.1–1.0 C.

Finally, we demonstrate the electrochemical performances of solid‐state Li||LiFePO_4_ cells based on cast‐CSE and ES‐CSE at 55 °C. All cells are subjected to measurements after activation treatment (Figure S15, Supporting Information). **Figure** **6a** presents the GCD curves of Li|ES‐CSE|LiFePO_4_ cell at 0.2 C, which exhibit typical charge and discharge plateaus at 3.45 V and a standard reversible specific capacity of 161.8 mAh g^−1^ LiFePO_4_.^[^ ^48^ ^]^ In addition, the voltage hysteresis between charge and discharge is as low as 80 mV, comparable to those of liquid electrolyte‐based cells. The GCD curves for the first 10 cycles are highly overlapped, indicating an excellent charge and discharge stability during cycling.

The long‐term cycling performance of cast‐CSE and ES‐CSE based solid‐state Li||LiFePO_4_ cells at 0.2 C is displayed in Figure 6b. The ES‐CSE‐based cell demonstrates a superior cycling stability for over 450 cycles with coulombic efficiency close to 100%, as the specific capacity is still as high as 136.3 mAh g^−1^ at the 450 cycle, corresponding to a capacity retention of 84%. While for the cast‐CSE‐based cell, the specific capacity fades rapidly from 159.2 for the first cycle to 104.7 mAh g^−1^ for the 180th cycle, and then a short circuit occurs.

Figure 6c shows the GCD curves of Li|ES‐CSE|LiFePO_4_ cell at 0.1 to 1 C. The voltage hysteresis between charge and discharge increases with current density, leading to a reduction in a reversible specific capacity. Rate performance (Figure 6d) shows that the reversible specific capacities of the ES‐CSE‐based cell are 160.4, 159.3, 155.3, 149.1, and 135.2 mAh g^−1^ at 0.1, 0.2, 0.5, 0.8, and 1 C, much higher than those of the cast‐CSE based cell.

**Figure 7:**
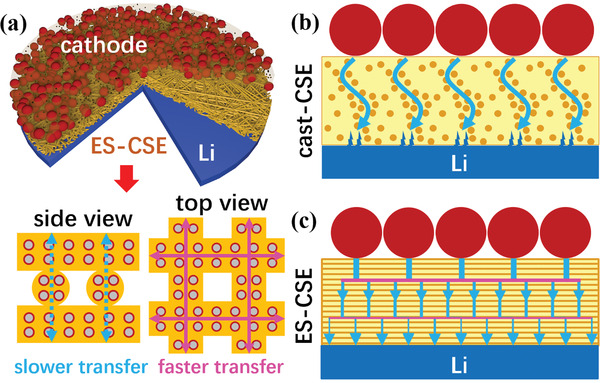
Schematic illustration of the superiority of ES‐CSE that works in solid‐state Li metal batteries. a) ES‐CSE in a solid‐state cell. b) Li‐ion transfer pathways in cast‐CSE. c) Li‐ion transfer pathways in ES‐CSE.

From all the results above, ES‐CSE with a unique structure (LLZO particles uniformly dispersed in interwoven PEO‐LiTFSI microfibers) is proved to be a very promising electrolyte for solid‐state Li metal batteries. As illustrated in **Figure** **7a**, the confinement of LLZO particles in PEO‐LiTFSI microfibers effectively avoids the severe agglomeration, which then improves the conductivity, transference number, and mechanical stability by stimulating the interaction between LLZO and PEO‐LiTFSI. In addition, the uniformly dispersed LLZO can also increase the apparent electrochemical stability of PEO‐LiTFSI by retarding the side reactions at the electrolyte/electrode interfaces. More importantly, the interwoven LLZO/PEO‐LiTFSI microfibers exhibit anisotropic Li‐ion conduction nature, that is, a much higher Li‐ion conductivity along the microfibers than that across the microfibers, which can significantly homogenize the electric field distribution during cycling and thus result in exceptional Li dendrite suppression capability. Figure 7b,c schematically illustrate the Li‐ion transfer pathways in the cast‐CSE and ES‐CSE based solid‐state Li metal batteries during charge, in which a more homogenous Li flux can be achieved in the ES‐CSE based cell due to the uniform LLZO dispersion and vertical/horizontal anisotropic Li‐ion conduction, leading to superior electrochemical performances. It should be noted here that vertically aligned CSE structures have been reported in previous literatures,^[^ ^32^, ^33^, ^34^, ^35^, ^36^ ^]^ which are designed for accelerating the Li‐ion transfer through CSEs by providing vertically continuous transfer channels. Apparently, these structures also demonstrate an anisotropic nature, that is, higher vertical conductivity and lower horizontal conductivity, which is opposite to our work. Nevertheless, we believe that these works are not contradictory to our work, because the main finding of our work is the even Li plating due to the effective homogenization of electric field distribution in ES‐CSE, rather than the improved Li‐ion conductivity.

## 3 Conclusion

In this work, ES‐CSE is successfully prepared by the coaxial electrospinning method, which consists of highly dispersed LLZO particles and interwoven PEO‐LiTFSI microfibers. ES‐CSE exhibits improved Li‐ion conduction properties, electrochemical stability, and mechanical stability, showing conductivity of 1.5 × 10^−4^ S cm^−1^ at 35 °C, a Li‐ion transference number of 0.49, an apparent electrochemical stability window of 0 to 4.1 V, and a tensile strength of 7.46 MPa. Cycling of Li symmetrical cells shows that ES‐CSE can suppress the Li dendrite at 0.1 mA cm^−2^ for over 1570 h. The unique vertical/horizontal anisotropic Li‐ion conduction is mainly responsible for the exceptional Li dendrite suppression capability, which can effectively homogenize the electric field distribution. Finally, ES‐CSE enables stable and fast cycling of solid‐state Li||LiFePO_4_ cells, as a specific capacity of 136.3 mAh g^−1^ can be achieved after 450 cycles at 0.2 C and 55 °C, corresponding to a capacity retention of 84%. This novel structure proposed by this study may also be applied in other CSEs for further improving the overall performances.

## 4 Experimental Section

### Materials Preparation

PEO (*M*_w_: 3×10^5^ g mol^−1^, Sigma‐Aldrich) and LiTFSI (Sigma‐Aldrich) was dried under vacuum at 60 and 100 °C for 24 h prior to use. Anhydrous ACN (Sigma‐Aldrich) was used as received. LLZO with the composition of Li_6.4_La_3_Zr_1.4_Ta_0.6_O_12_ were synthesized by solid‐state reaction,^[^ ^49^ ^]^ and then high‐energy ball‐milled. PEO/LiTFSI and cast‐CSE were prepared by tape casting the PEO/LiTFSI/ACN solution and LLZO/PEO/LiTFSI/ACN solution with EO/Li ratio of 8 and then dried in a vacuum oven at 50 °C for 48 h. ES‐CSE (or AES‐CSE) was prepared by electrospinning with the LLZO/PEO/LiTFSI/ACN as core solution and PEO/LiTFSI/ACN as shell solution under 17 kV using a drum collector rotating at 60 rpm (or 800 rpm) and then dried in a vacuum oven at 50 °C for 48 h. The LLZO contents in the core solutions were 15 to 48 wt%.

### Structure Characterizations

XRD measurements were performed on Rigaku Ultima IV at a scan rate of 5° min^−1^. SEM images were collected by FEI quanta FEG 450. TEM observations were conducted on JEOL JEM‐2100F. FTIR spectra were recorded by Bruker VERTEX 70 in transmission mode. XPS tests were performed on ThermoFisher Thermo Scientific K‐Alpha+. DSC measurements were conducted on a TA Q5000IR with a heating rate of 5 °C min^−1^ under N_2_ atmosphere. Stress‐strain curves were obtained using MTS CMT6103.

### Electrochemical Measurements

Solid‐state cells were all assembled in CR2016. The conductivities of the electrolytes were calculated from the EIS results of steel|electrolyte|steel cells using Gamry Interface 1000E. The electronic and Li‐ion transference numbers of the electrolytes were respectively tested in steel|electrolyte|steel cells and Li|electrolyte|Li cells using DC polarization (1 V for electronic transference number tests and 10 mV for Li‐ion transference number tests) and EIS before and after polarization. The calculation formula is:

(2) $$T_{\mathrm{Li}-\mathrm{ion}}=\frac{I_{s}}{I_{0}}\frac{\left(\Delta V-I_{0}R_{0}\right)}{\left(\Delta V-I_{s}R_{s}\right)}$$

where *R*_s_ and *R*_0_ are the interfacial resistances acquired from the diameters of the second semicircles in EIS before and after polarization, respectively, *I*_0_ is the initial current, *I*_s_ is the steady‐state current, and Δ*V* is the applied voltage. The electrochemical stability windows of the electrolytes were evaluated by LSV curves of Li|electrolyte|steel cells using CHI660E with a scan rate of 5 mV s^−1^. Li dendrite suppression capabilities of the electrolytes were measured by GCD of Li|electrolyte|Li cells using Land CT2001A with the charge/discharge duration of 30/30 min. In the orientational Li stripping and plating experiments (Figure S16, Supporting Information), the DC resistance was obtained by a digital multimeter (Victor VC890C^+^), the temperature was controlled by a heating holder (IKA C‐MAG HS 4), and the orientational electrochemical Li plating was driven by a programmable DC power supply (Maynuo M8811). Li|electrolyte|LiFePO_4_ cells were assembled using LiFePO_4_/PVDF/super‐P as cathode and Li metal as anode. The electrolyte/cathode interface optimization was achieved by adding molten PEO‐LiTFSI onto the cathode surface.^[^ ^35^ ^]^ GCD of the Li|electrolyte|LiFePO_4_ cells were conducted on Land CT2001A.

### Statistical Analysis

The statistics of the microfiber diameters were performed using Gaussian function by Origin 9.0 software and the sample size was 30.

## Conflict of Interest

The authors declare no conflict of interest.

## Supporting information

Supporting Information

## Data Availability

Research data are not shared.

## References

[1] J.‐M. Tarascon, M. Armand, Nature 2001, 414, 359. 11713543 10.1038/35104644

[2] H. Li, Z. Wang, L. Chen, X. Huang, Adv. Mater. 2009, 21, 4593.

[3] B. Dunn, H. Kamath, J.‐M. Tarascon, Science 2011, 334, 928. 22096188 10.1126/science.1212741

[4] S. Zhang, H. Gu, H. Pan, S. Yang, W. Du, X. Li, M. Gao, Y. Liu, M. Zhu, L. Ouyang, D. Jian, F. Pan, Adv. Energy Mater. 2016, 7, 1601066.

[5] A. Manthiram, X. Yu, S. Wang, Nat. Rev. Mater. 2017, 2, 16103.

[6] S. Xia, X. Wu, Z. Zhang, Y. Cui, W. Liu, Chem 2018, 5, 753.

[7] M. Zhu, Y. Pang, F. Lu, X. Shi, J. Yang, S. Zheng, ACS Appl. Mater. Interfaces 2019, 11, 14136. 30907580 10.1021/acsami.9b01326

[8] C. Wang, T. Wang, L. Wang, Z. Hu, Z. Cui, J. Li, S. Dong, X. Zhou, G. Cui, Adv. Sci. 2019, 6, 1901036. 10.1002/advs.201901036 PMC6865005 31763139

[9] Y. Pang, X. Wang, X. Shi, F. Xu, L. Sun, J. Yang, S. Zheng, Adv. Energy Mater. 2020, 10, 1902795.

[10] K. J. Kim, M. Balaish, M. Wadaguchi, L. Kong, J. L. M. Rupp, Adv. Energy Mater. 2021, 11, 2002689.

[11] Y. Yan, J. Ju, S. Dong, Y. Wang, L. Huang, L. Cui, F. Jiang, Q. Wang, Y. Zhang, G. Cui, Adv. Sci. 2021, 8, 2003887. 10.1002/advs.202003887 PMC8097327 33977057

[12] Z. Gao, H. Sun, L. Fu, F. Ye, Y. Zhang, W. Luo, Y. Huang, Adv. Mater. 2018, 30, 1705702. 10.1002/adma.201705702 29468745

[13] Y. Pang, j. Pan, j. Yang, s. Zheng, c. Wang, Electrochem. Energy Rev. 2021, 10.1007/s41918-020-00092-1.

[14] W. H. Meyer, Adv. Mater. 1998, 10, 439. 21647973 10.1002/(SICI)1521-4095(199804)10:6<439::AID-ADMA439>3.0.CO;2-I

[15] Y. Zhao, L. Wang, Y. Zhou, Z. Liang, N. Tavajohi, B. Li, T. Li, Adv. Sci. 2021, 8, 2003675. 10.1002/advs.202003675 PMC8025011 33854893

[16] W. Liu, D. Lin, J. Sun, G. Zhou, Y. Cui, ACS Nano 2016, 10, 11407. 28024352 10.1021/acsnano.6b06797

[17] X. Zhang, T. Liu, S. Zhang, X. Huang, B. Xu, Y. Lin, B. Xu, L. Li, C.‐W. Nan, Y. Shen, J. Am. Chem. Soc. 2017, 139, 13779. 28898065 10.1021/jacs.7b06364

[18] D. Li, L. Chen, T. Wang, L. Z. Fan, ACS Appl. Mater. Interfaces 2018, 10, 7069. 29411972 10.1021/acsami.7b18123

[19] J. Bae, Y. Li, F. Zhao, X. Zhou, Y. Ding, G. Yu, Energy Storage Mater. 2018, 15, 46.

[20] P. Zhu, C. Yan, M. Dirican, J. Zhu, J. Zang, R. K. Selvan, C.‐C. Chung, H. Jia, Y. Li, Y. Kiyak, N. Wu, X. Zhang, J. Mater. Chem. A 2018, 6, 4279.

[21] L. Chen, W. Li, L. Z. Fan, C. W. Nan, Q. Zhang, Adv. Funct. Mater. 2019, 29, 1901047.

[22] S. Tang, W. Guo, Y. Fu, Adv. Energy Mater. 2020, 11, 2000802.

[23] H. Chen, D. Adekoya, L. Hencz, J. Ma, S. Chen, C. Yan, H. Zhao, G. Cui, S. Zhang, Adv. Energy Mater. 2020, 10, 2000049.

[24] T. Jiang, P. He, G. Wang, Y. Shen, C. W. Nan, L. Z. Fan, Adv. Energy Mater. 2020, 10, 1903376.

[25] K. Pan, L. Zhang, W. Qian, X. Wu, K. Dong, H. Zhang, S. Zhang, Adv. Mater. 2020, 32, 2000399. 10.1002/adma.202000399 32173931

[26] S. Li, S.‐Q. Zhang, L. Shen, Q. Liu, J.‐B. Ma, W. Lv, Y.‐B. He, Q.‐H. Yang, Adv. Sci. 2020, 7, 1903088. 10.1002/advs.201903088 PMC7055568 32154083

[27] J. Zhang, N. Zhao, M. Zhang, Y. Li, P. K. Chu, X. Guo, Z. Di, X. Wang, H. Li, Nano Energy 2016, 28, 447.

[28] Z. Li, H. M. Huang, J. K. Zhu, J. F. Wu, H. Yang, L. Wei, X. Guo, *ACS Appl. Mater. Interfaces* 2019, 11, 784. 10.1021/acsami.8b17279 30525410

[29] Z. Wan, D. Lei, W. Yang, C. Liu, K. Shi, X. Hao, L. Shen, W. Lv, B. Li, Q.‐H. Yang, F. Kang, Y.‐B. He, Adv. Funct. Mater. 2019, 29, 1805301.

[30] H. Huo, Y. Chen, J. Luo, X. Yang, X. Guo, X. Sun, Adv. Energy Mater. 2019, 9, 1804004.

[31] K. K. Fu, Y. h. Gong, J. q. Dai, A. Gong, X. g. Han, Y. g. Yao, C. w. Wang, Y. b. Wang, Y. Chen, C. y. Yan, Y. Li, E. D. Wachsman, L. b. Hu, Proc. Natl. Acad. Sci. U. S. A. 2016, 113, 7094. 27307440 10.1073/pnas.1600422113 PMC4932948

[32] Z. Li, W.‐X. Sha, X. Guo, ACS Appl. Mater. Interfaces 2019, 11, 26920. 31268655 10.1021/acsami.9b07830

[33] W. Tang, S. Tang, X. Guan, X. Zhang, Q. Xiang, J. Luo, Adv. Funct. Mater. 2019, 29, 1900648.

[34] H. Zhai, P. Xu, M. Ning, Q. Cheng, J. Mandal, Y. Yang, Nano Lett. 2017, 17, 3182. 28409638 10.1021/acs.nanolett.7b00715

[35] X. Wang, H. Zhai, B. Qie, Q. Cheng, A. Li, J. Borovilas, B. Xu, C. Shi, T. Jin, X. Liao, Y. Li, X. He, S. Du, Y. Fu, M. Dontigny, K. Zaghib, Y. Yang, Nano Energy 2019, 60, 205.

[36] J. Wan, J. Xie, X. Kong, Z. Liu, K. Liu, F. Shi, A. Pei, H. Chen, W. Chen, J. Chen, X. Zhang, L. Zong, J. Wang, L.‐Q. Chen, J. Qin, Y. Cui, Nat. Nanotechnol. 2019, 14, 705. 31133663 10.1038/s41565-019-0465-3

[37] X. Zhang, J. Xie, F. Shi, D. Lin, Y. Liu, W. Liu, A. Pei, Y. Gong, H. Wang, K. Liu, Y. Xiang, Y. Cui, Nano Lett. 2018, 18, 3829. 29727578 10.1021/acs.nanolett.8b01111

[38] W. Liu, S. W. Lee, D. Lin, F. Shi, S. Wang, A. D. Sendek, Y. Cui, Nat. Energy 2017, 2, 17035.

[39] S. Agarwal, J. H. Wendorff, A. Greiner, Adv. Mater. 2009, 21, 3343. 20882501 10.1002/adma.200803092

[40] J. Yoon, H.‐S. Yang, B.‐S. Lee, W.‐R. Yu, Adv. Mater. 2018, 30, 1704765. 10.1002/adma.201704765 30152180

[41] W. Liu, N. Liu, J. Sun, P.‐C. Hsu, Y. Li, H.‐W. Lee, Y. Cui, Nano Lett. 2015, 15, 2740. 25782069 10.1021/acs.nanolett.5b00600

[42] F. Lu, Y. Pang, M. Zhu, F. Han, J. Yang, F. Fang, D. Sun, S. Zheng, C. Wang, Adv. Funct. Mater. 2019, 29, 1809219.

[43] X. Shi, Y. Pang, B. Wang, H. Sun, X. Wang, Y. Li, J. Yang, H.‐W. Li, S. Zheng, Mater. Today Nano 2020, 10, 100079.

[44] J. Liang, S. Hwang, S. Li, J. Luo, Y. Sun, Y. Zhao, Q. Sun, W. Li, M. Li, M. N. Banis, X. Li, R. Li, L. Zhang, S. Zhao, S. Lu, H. Huang, D. Su, X. Sun, Nano Energy 2020, 78, 105107.

[45] C. Xu, B. Sun, T. Gustafsson, K. Edström, D. Brandell, M. Hahlin, J. Mater. Chem. A 2014, 2, 7256.

[46] L. Chen, Y. Li, S.‐P. Li, L.‐Z. Fan, C.‐W. Nan, J. B. Goodenough, Nano Energy 2018, 46, 176.

[47] L. Zhang, T. Yang, C. Du, Q. Liu, Y. Tang, J. Zhao, B. Wang, T. Chen, Y. Sun, P. Jia, H. Li, L. Geng, J. Chen, H. Ye, Z. Wang, Y. Li, H. Sun, X. Li, Q. Dai, Y. Tang, Q. Peng, T. Shen, S. Zhang, T. Zhu, J. Huang, Nat. Nanotechnol. 2020, 15, 94. 31907440 10.1038/s41565-019-0604-x

[48] Y. Lin, M. X. Gao, D. Zhu, Y. F. Liu, H. G. Pan, J. Power Sources 2008, 184, 444.

[49] Y. Li, Y. Cao, X. Guo, Solid State Ionics 2013, 253, 76.

