# Supplementary material for: Uniform and Anisotropic Solid Electrolyte Membrane Enables Superior Solid‐State Li Metal Batteries
Source: Adv Sci (Weinh). 2021 Jun 2;8(16):2100899. doi: 10.1002/advs.202100899 (PMC8373100; doi:10.1002/advs.202100899)
Supplement: Supplementary file 1 — Supporting Information [file ADVS-8-2100899-s001.pdf]

WILEY-VCH

© 2021 Wiley-VCH GmbH

ADVANCED SCIENCE
Open Access

Supporting Information

for *Adv. Sci.*, DOI: 10.1002/advs.202100899

Uniform and Anisotropic Solid Electrolyte Membrane Enables Superior Solid-State Li Metal Batteries

Zumin Guo, Yuepeng Pang, Shuixin Xia, Fen Xu, Junhe Yang, Lixian Sun*, Shiyou Zheng**

Supporting Information

Uniform and Anisotropic Solid Electrolyte Membrane Enables Superior Solid-State Li Metal Batteries

Zumin Guo, Yuepeng Pang, Shuixin Xia, Fen Xu, Junhe Yang, Lixian Sun*, Shiyou Zheng**

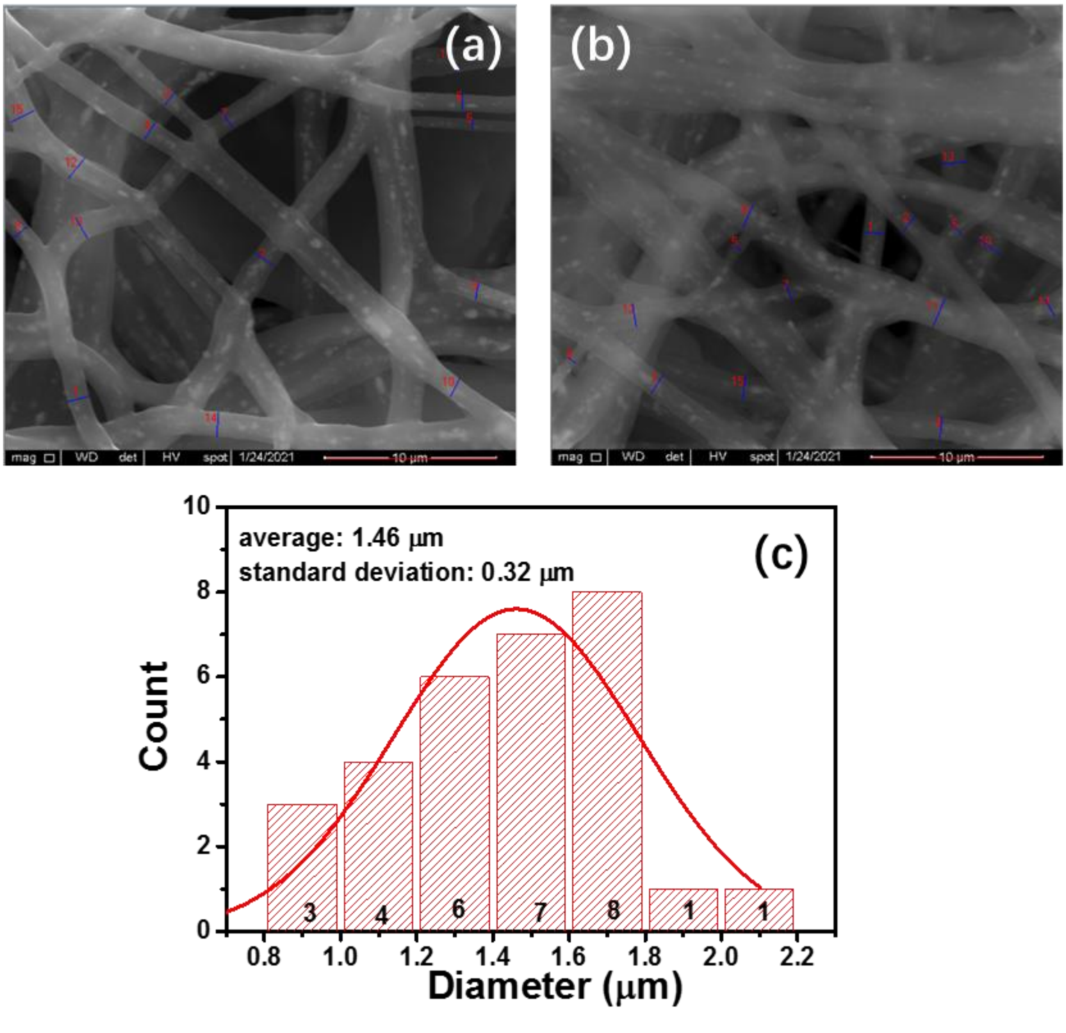

Figure S1. Statistics of the microfiber diameters of ES-CSE.

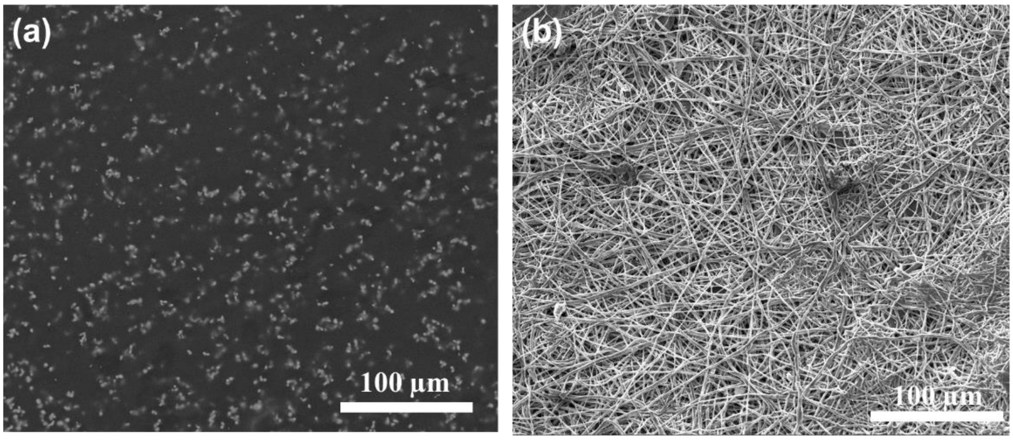

Figure S2. Top-view SEM images of cast-CSE (a) and ES-CSE (b).

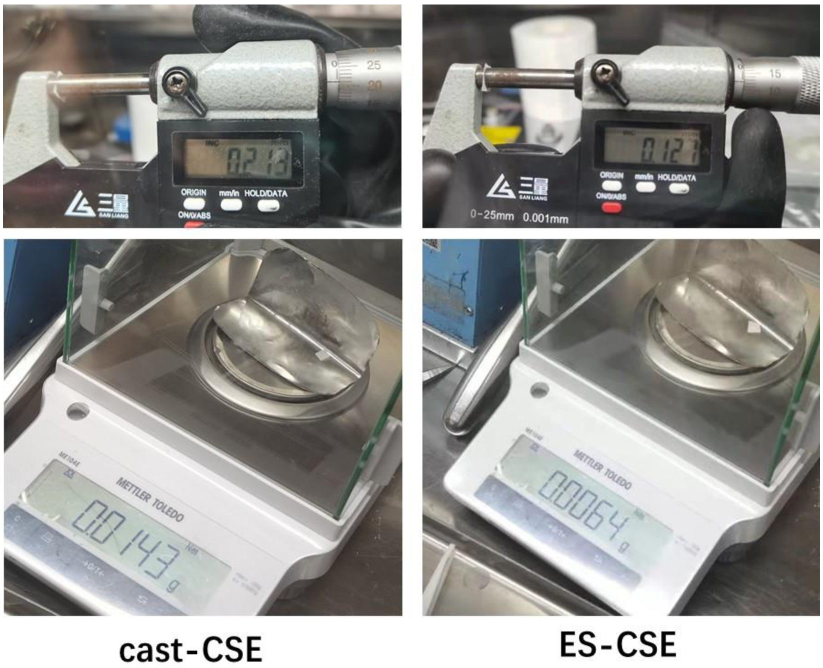

Figure S3. Thicknesses and weights of cast-CSE (a, b) and ES-CSE (c, d) 8 × 8 mm^2 square membranes.

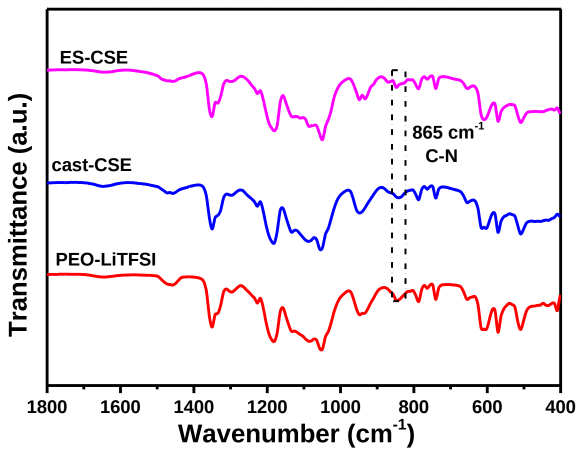

Figure S4. FTIR spectra of PEO-LiTFSI, cast-CSE and ES-CSE.

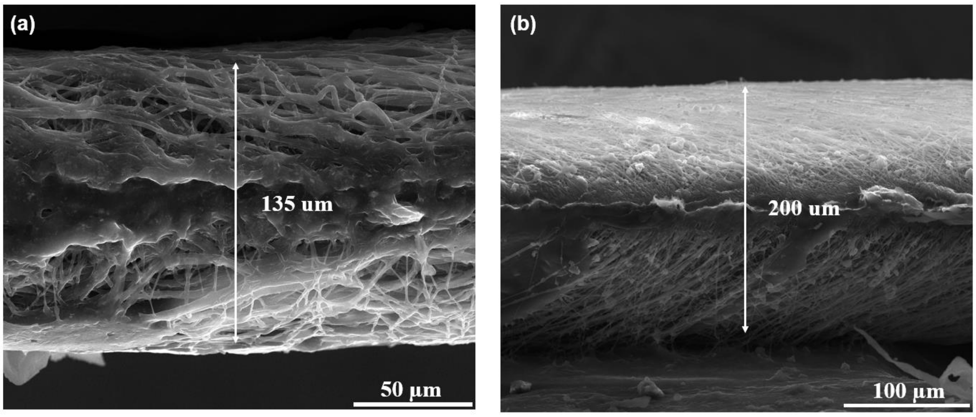

Figure S5. Side-view SEM images of ES-CSE with different electrospinning durations. (a) 5h. (b) 8h.

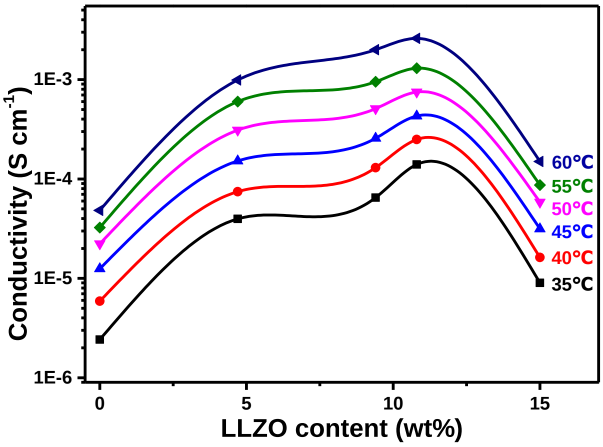

Figure S6. Conductivities of ES-CSE with different LLZO contents at different temperatures.

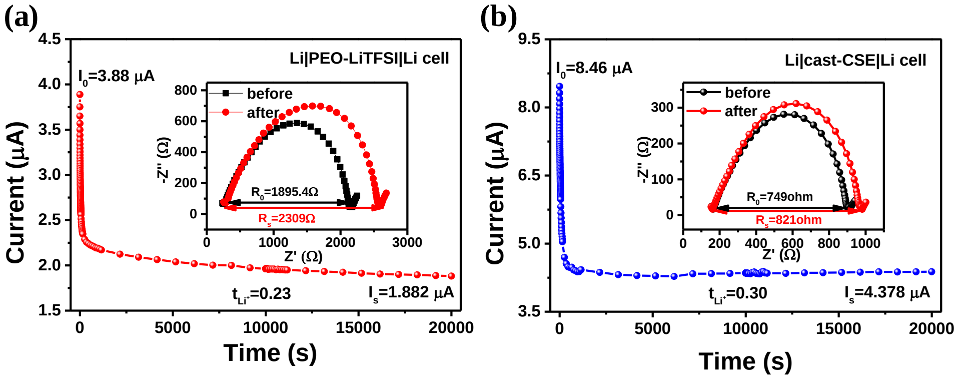

Figure S7. Li-ion transference numbers of PEO-LiTFSI (a) and cast-CSE (b).

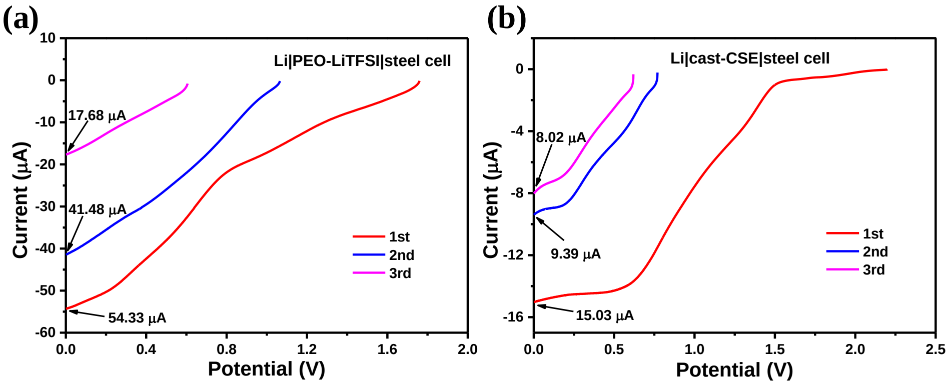

Figure S8. Repeated LSV curves of PEO-LiTFSI (a) and cast-CSE (b) from OCV to 0 V.

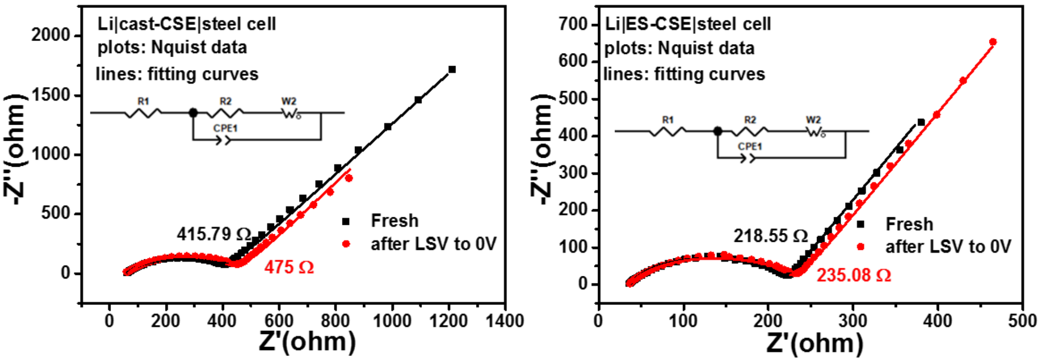

Figure S9. EIS of cast-CSE and ES-CSE based Li||steel cells before and after LSV to 0V.

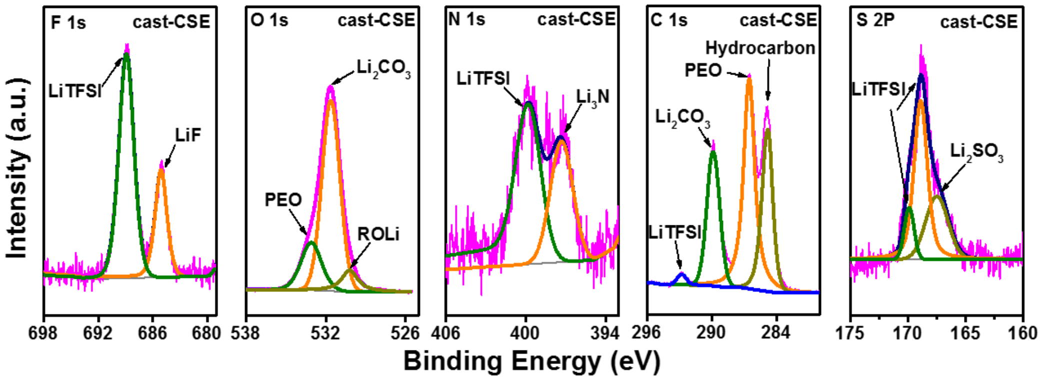

Figure S10. XPS results of the in-situ formed SEI on the steel side for cast-CSE after repeated scanning from OCV to 0 V.

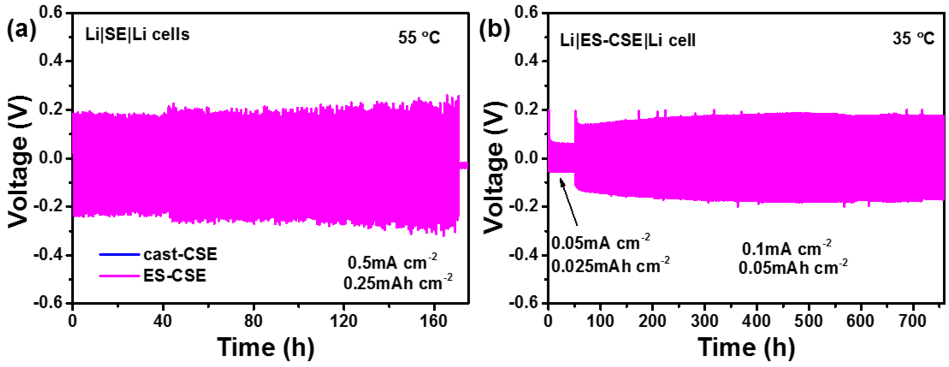

Figure S11. GCD profiles of Li|ES-CSE|Li cell at 0.5 mA cm^{-2} and 55 ºC (a), 0.1 mA cm^{-2} and 35 ºC (b).

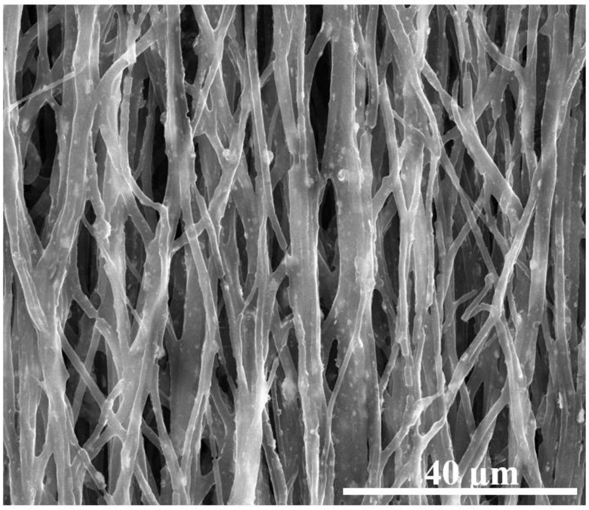

Figure S12. Top-view SEM images of AES-CSE.

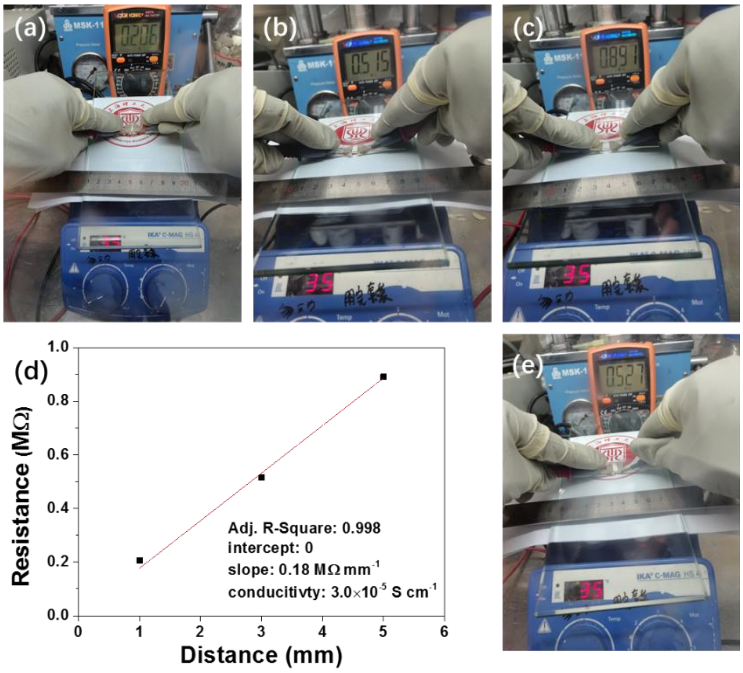

Figure S13. (a, b, c) Optical photographs of the horizontal Li-ion conduction experiments using different distances of 1, 3, and 5 mm. (d) Linear fitting of the resistance versus distance plots. (e) Optical photographs of the horizontal Li-ion conduction experiments using rectangular Li electrodes.

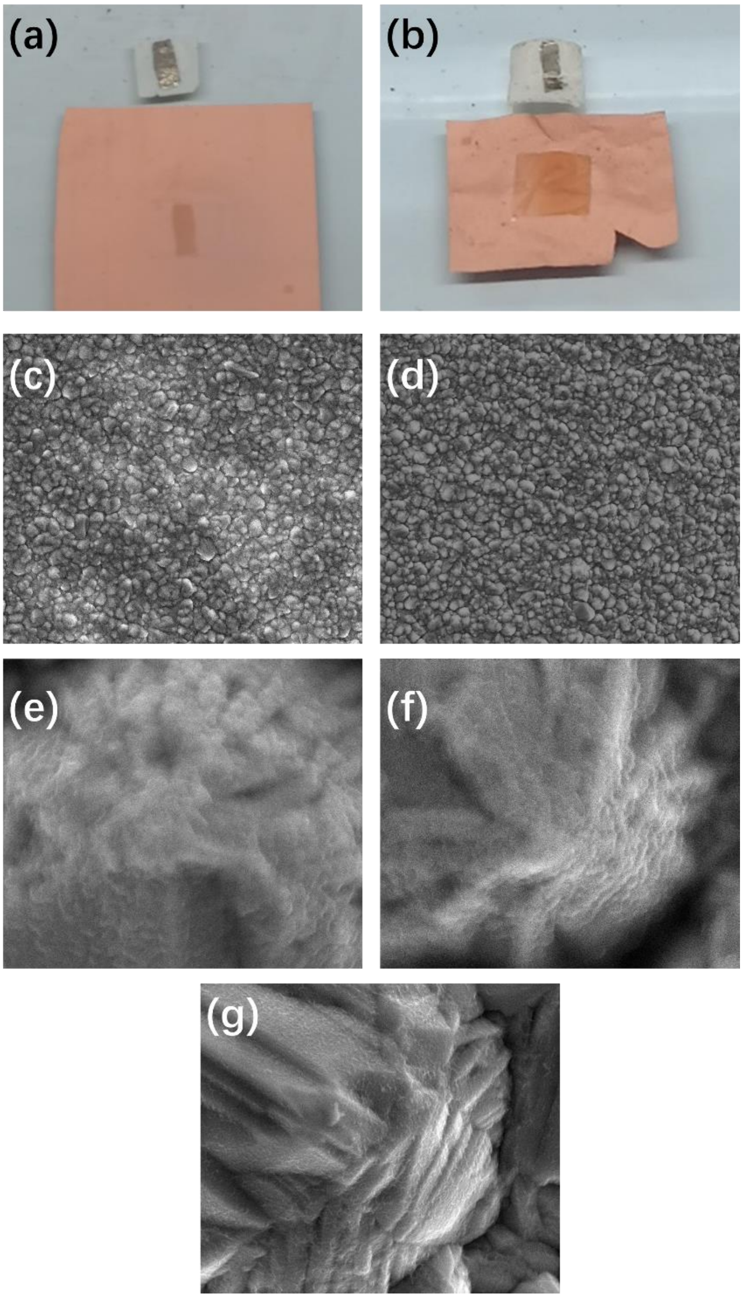

Figure S14. Optical photographs (a, b), SEM images (c, d) and high-resolution SEM images (e, f) of Cu foil after electrochemical Li plating through cast-CSE and ES-CSE. (g) High-resolution SEM image of fresh Cu foil.

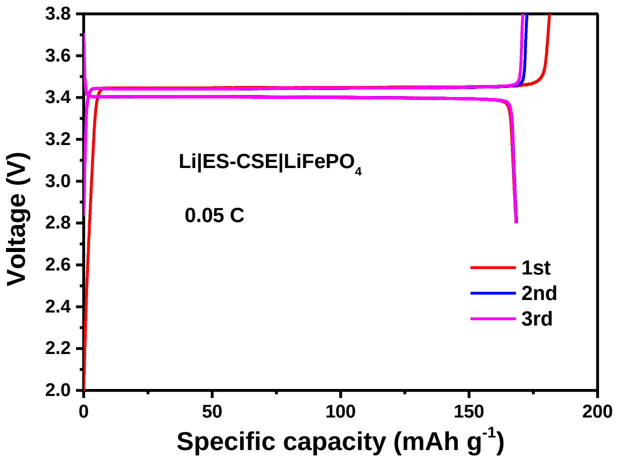

Figure S15. GCD profiles of Li|ES-CSE|$LiFePO_4$ cell during the activation treatment.

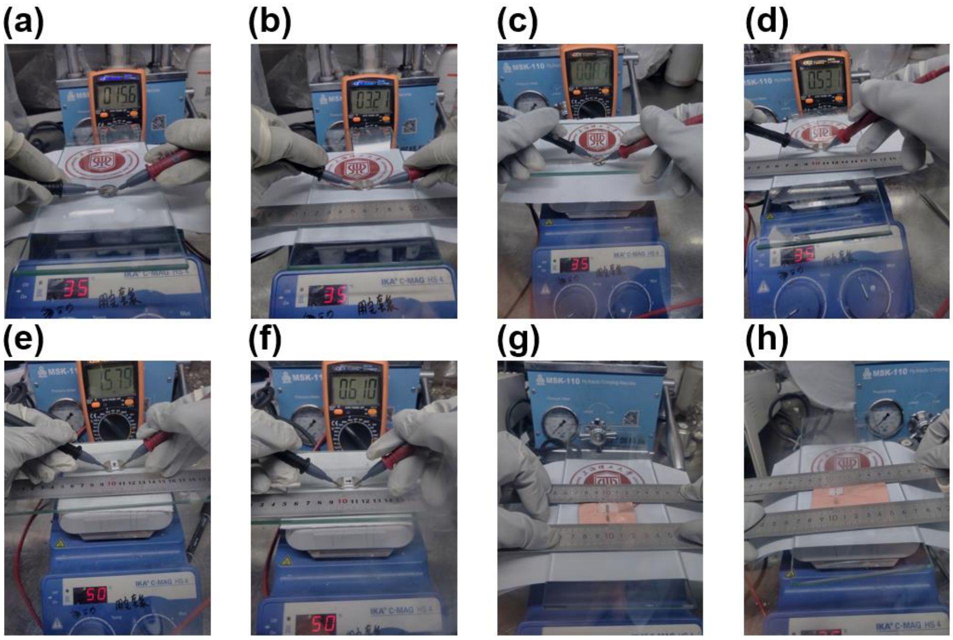

Figure S16. (a-h) Optical photographs of the corresponding vertical/horizontal anisotropic Li-ion conduction experiments.
